# An evaluation of strategies to achieve greater than 90% coverage of maternal influenza and pertussis vaccines including an economic evaluation

**DOI:** 10.1186/s12884-021-04248-9

**Published:** 2021-11-15

**Authors:** Michelle L. Giles, Kong Khai, Sushena Krishnaswamy, Karen Bellamy, Margaret Angliss, Christopher Smith, Olivia Fay, Paul Paddle, Beverley Vollenhoven

**Affiliations:** 1 Department of Obstetrics and Gynaecology, Monash University, 246 Clayton Road, Clayton, 3168 Australia; 2 Women’s and Newborn Program, Monash Health, 246 Clayton Road, Clayton, Victoria Australia; 3 Department of Infectious Diseases, Monash Health, 246 Clayton Road, Clayton, Victoria Australia; 4 Monash Immunisation, Monash Health, 246 Clayton Road, Clayton, Victoria Australia; 5 Aspex Consulting, 212 Clarendon Street, East Melbourne, Victoria Australia; 6 Department of Surgery, Monash University, 246 Clayton Road, Clayton, Victoria Australia

**Keywords:** Immunisation, Vaccine, Influenza, Pertussis, Barriers, Service delivery

## Abstract

**Background:**

Maternal immunisation is an essential public health intervention aimed at improving the health outcomes for pregnant women and providing protection to the newborn. Despite international recommendations, safety and efficacy data for the intervention, and often a fully funded program, uptake of vaccines in pregnancy remain suboptimal. One possible explanation for this includes limited access to vaccination services at the point of antenatal care. The aim of this study is to evaluate the change in vaccine coverage among pregnant women following implementation of a modified model of delivery aimed at improving access at the point of antenatal care, including an economic evaluation.

**Methods:**

This prospective multi-centre study, using action research design, across six maternity services in Victoria, Australia, evaluated the implementation of a co-designed vaccine delivery model (either a pharmacy led model, midwife led model or primary care led model) supported by provider education. The main outcome measure was influenza and pertussis vaccine uptake during pregnancy and the incremental cost of the new model (compared to existing models) and the cost-effectiveness of the new model at each participating health service.

**Results:**

Influenza vaccine coverage in 2019 increased between 50 and 196% from baseline. All services reduced their average cost per immunisation under the new platforms due to efficiencies achieved in the delivery of maternal immunisations. This cost saving ranged from $9 to $71.

**Conclusion:**

Our study demonstrated that there is no ‘one size fits all’ model of vaccine delivery. Future successful strategies to improve maternal vaccine coverage at other maternity services should be site specific, multifaceted, targeted at the existing barriers to maternal vaccine uptake, and heavily involve local stakeholders in the design and implementation of these strategies. The cost-effectiveness analysis indicates that an increase in maternal influenza immunisation uptake can be achieved at a relatively modest cost through amendment of maternal immunisation platforms.

## Background

Influenza infection can cause significant illness in pregnant women and can also adversely affect fetal and neonatal outcomes [1–5]. Influenza vaccination during pregnancy is a safe and effective strategy to protect both pregnant women and their infants from influenza infection [6–8] and is recommended by the World Health Organisation (WHO) for all pregnant women. Similarly, maternal pertussis vaccination has a vaccine effectiveness of at least 91% in protecting infants younger than 3 months of age against pertussis infection [9, 10] and in many high and middle-income countries, pertussis containing vaccine is also recommended for all pregnant women.

Despite these recommendations, maternal vaccine coverage among pregnant women remains low. In many high-income countries where influenza vaccine is recommended, vaccine coverage remains suboptimal, with reported coverage of 49% in the United States in 2018, 44% in the United Kingdom from 2019 to 2020, and 67% in Australia in 2018 [11–13]. Likewise in countries recommending pertussis vaccination during pregnancy, vaccine coverage has ranged between 44% in the United Kingdom to 78% in New Zealand [14, 15]. These figures have been stubbornly resistant to change despite accumulating safety and efficacy data.

At the health service level, women’s access to vaccines has been demonstrated to influence coverage [16–18]. By integrating maternal vaccination into routine antenatal care, pregnant women can access vaccines more readily. This may in turn improve vaccine coverage, as shown in several studies [16–18]. Several strategies to improve vaccine coverage have been reported in the literature. These include strategies to increase pregnant women’s awareness of the effectiveness and safety of the vaccines, strategies to facilitate healthcare provider (HCP) recommendation of vaccination in pregnancy, and strategies to improve access [19].

In Australia, influenza and pertussis vaccines have been available at no cost to pregnant women since 2010 and 2015 respectively. Immunisation status for each pregnant woman giving birth in both public and private maternity services in Victoria has been routinely submitted to the Department of Health and Human Services as part of the Victorian Perinatal Data Collection since 2015 [13]. In 2017, the state-wide maternal influenza vaccine coverage was 54% [20]. This was lower than the maternal pertussis vaccination coverage in Victoria, which was 78% [20].

To improve coverage of maternal vaccines overall and reduce disparity between services across Victoria, a multi-centre study was designed with the aim of increasing access to maternal vaccines at the point of antenatal care. After introducing modified or new models for vaccine delivery at participating services, vaccine coverage in 2019 amongst pregnant women giving birth at these services was compared to vaccine coverage in 2018. In addition to comparing vaccine coverage, the study included an economic evaluation which assessed the incremental cost of the new models, compared to existing models, and the cost-effectiveness of the new models at each participating health service.

## Methods

**Fig. 1:**
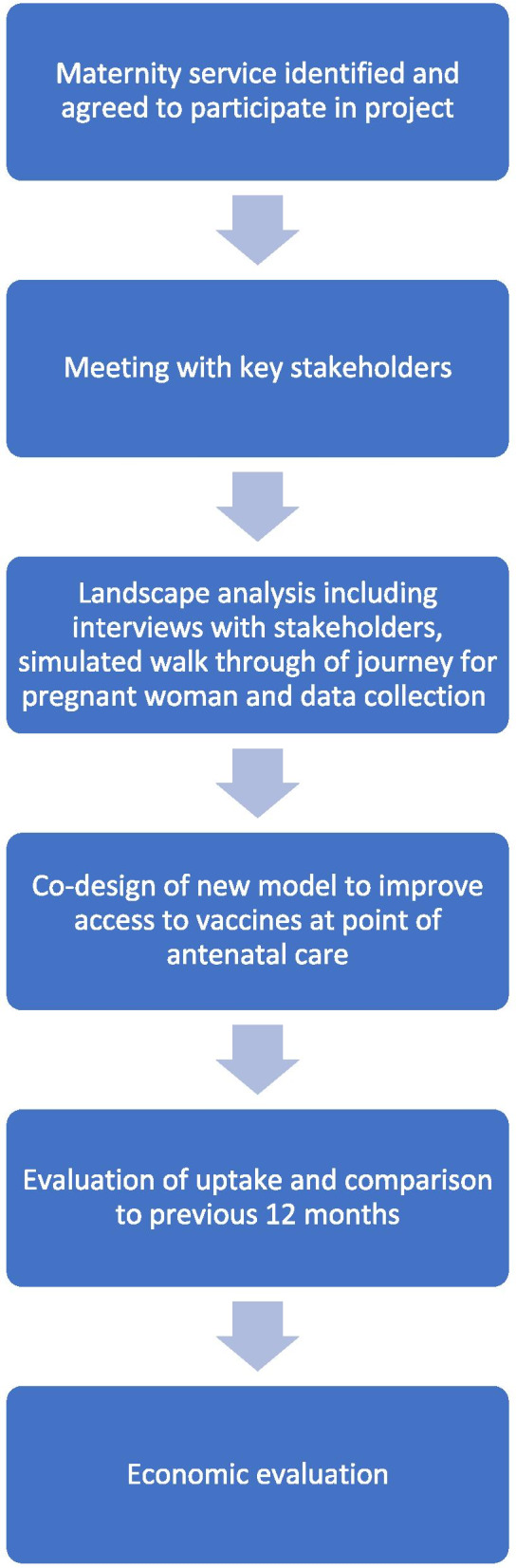
An overview of the steps in the project

This project used action research design methodology. This involves collecting information regarding the current program and outcomes, analysing the information, developing a plan with key stakeholders to improve the outcomes, implementing the plan, collecting data during and post implementation and developing conclusions regarding the changes. An overview of the key steps in the project methodology is summarised in Fig. 1. Seven services were approached to participate in the project. A range of services were invited to represent both metropolitan and regional maternity services, a range of maternity care capability, and with a range of vaccine coverage. One service declined due to concerns about being able to commit adequate time to the project given other workforce responsibilities. Vaccine coverage at baseline for each site was available from the Victorian perinatal services performance indicators report [20].

Once maternity services agreed to participate in the project, key stakeholders (midwives, obstetricians, pharmacists, infection control practitioners, representatives from the quality unit, paediatricians, hospital executives) at each site were invited to participate in a landscape analysis of their service. This landscape analysis aimed to:

(i) Assess the existing model(s) of maternal vaccine delivery (the existing models included referral to the GP for vaccine or midwife administered)
(ii) Identify barriers and facilitators for delivering maternal vaccines

The landscape analyses were conducted between November and December 2018. The landscape analyses involved collecting data from interviews with stakeholders and conducting a simulated “walk through” of the process by which vaccines are recommended and/or delivered to pregnant women at each service. Data collected included:

- Existing policies and procedures regarding maternal immunisation
- Methods utilised to create demand for maternal vaccination (e.g. posters in the waiting room, information booklets)
- Capacity for procurement and storage of vaccines, and maintenance of cold chain onsite
- Current systems for recording vaccinations received by women
- Staff education and training (specific to immunisation)

Based on the findings from the landscape analysis and in collaboration with local stakeholders at each site, the existing model of vaccine delivery was reviewed to overcome identified barriers to vaccine delivery. A modified model for delivering maternal vaccines was implemented at each site before the 2019 seasonal influenza vaccine became available in April 2019. Education for staff about maternal immunisation was provided at each site. This education comprised of a face-to-face teaching session with maternity care providers. Frequently asked questions (FAQs) on influenza and pertussis vaccines for health care providers were developed and provided to each site as were posters promoting vaccines in pregnancy. Booklets with information about influenza vaccine in pregnancy were produced and provided to each service to be distributed to pregnant women. For the sites expanding midwife led pertussis vaccination to include influenza vaccination, the project facilitated and supported nurse immuniser training for the development of a “vaccine champion”.

In order to undertake the cost-effectiveness analysis, the following inputs were used; immunisation activity for each of the participating health services, including the total number of women who gave birth at each of the participating health services and the proportion immunized against influenza, across the baseline and evaluation periods. Costs incurred by participating health services were collected through a cost survey completed by the participating health services. The survey captured costs incurred relating to capital, non-capital and variable costs.

Using the above inputs, the following economic analysis was undertaken:

- Assessment of the incremental cost of the new models at each participating health service; and
- Cost-effectiveness analysis comparing the:
- Average cost per maternal immunisation under the existing and new models at each participating health service; and
- Incremental Cost Effectiveness Ratio’s (ICER) of the new models. This allowed for the cost-effectiveness of the new models to be compared. That is, of the new models, which model had the lower additional cost per additional immunisation.

## Results

In 2018, pertussis vaccine coverage ranged from 60 to 97% and influenza vaccine coverage ranged from 34 to 56% across the six maternity services. Key characteristics of each maternity service and the existing model of vaccine delivery is summarised in Table 1.

**Table 1:** Characteristics and 2017 vaccine coverage

Maternity service	Capability Level	Distance from Melbourne CBD in kms	Pertussis vaccine coverage (2017)	Influenza vaccine coverage (2017–2018)^**a**^	Influenza coverage (2018–2019)^**b**^	Existing pathway of pertussis vaccine delivery (2018)	Existing pathway of influenza vaccine delivery (2018)	Primary Intervention
A	6*	< 20	60%	46%	57%	Refer to GP	Refer to GP	Publicprivate pharmacy model
B	5^	100–200	91%	47%	61%	Refer to GP	Refer to GP	Education and monthly audit
C	4^	200–300	88%	56%	75%	Midwives giving some but refer to GP mainly	Refer to GP	Midwife led model
D	4^	200–300	77%	34%	49%	Midwives giving vaccines	Refer to GP	Midwife led model
E	3^#^	20–100	97%	28%	62%	Midwives giving vaccines	Refer to GP	Midwife led model
F	2^#^	300–400	97%	27%	32%	Midwives giving vaccines	Refer to GP	Midwife led model

Capability level: *complex pregnancies, births and neonatal intensive care

^medium complexity pregnancies and babies. Management of labour, birth and puerperium at 34 weeks (level 4) or 32 weeks (level 5)

^#^low complexity pregnancies and babies from 37 weeks gestation

^a^Victorian perinatal services performance indicators 2017–2018

^b^Victorian perinatal services performance indicators 2018–2019

Four maternity services with pre-existing midwife led pertussis vaccination delivery decided to expand this model to include influenza vaccine. One maternity service introduced a private-public model utilising a co-located private pharmacy and one maternity service, after the landscape analysis and key stakeholder meetings decided not to change their model of vaccine delivery continuing to refer to general practitioners (GPs) for vaccine administration. Additional strategies introduced at individual maternity services (at their own discretion) included a health service specific educational video about maternal influenza vaccine sent by text message (Health Service B and C, Table 1), changes to documentation requirements such that documentation at the time of administration rather than post partum was made mandatory (Health service D, E and F Table 1), and monthly audits of performance were introduced (Health service B, Table 1).

**Fig. 2:**
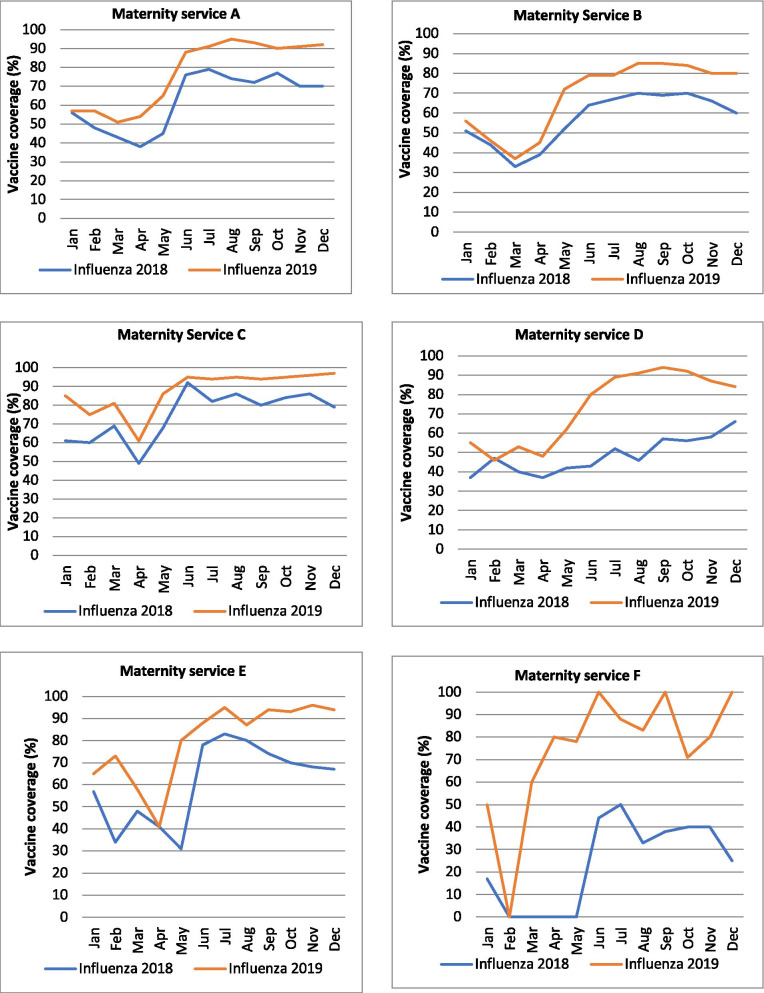
Maternal influenza vaccine coverage per month (2018 compared to 2019) for each maternity service

Influenza vaccine coverage in 2019 increased between 50 and 196% from baseline. The monthly influenza vaccine coverage in 2019 compared to the monthly vaccine coverage in 2018 is summarised in Fig. 2. The increment in vaccine coverage was sustained until December 2019 in all six maternity services.

**Fig. 3:**
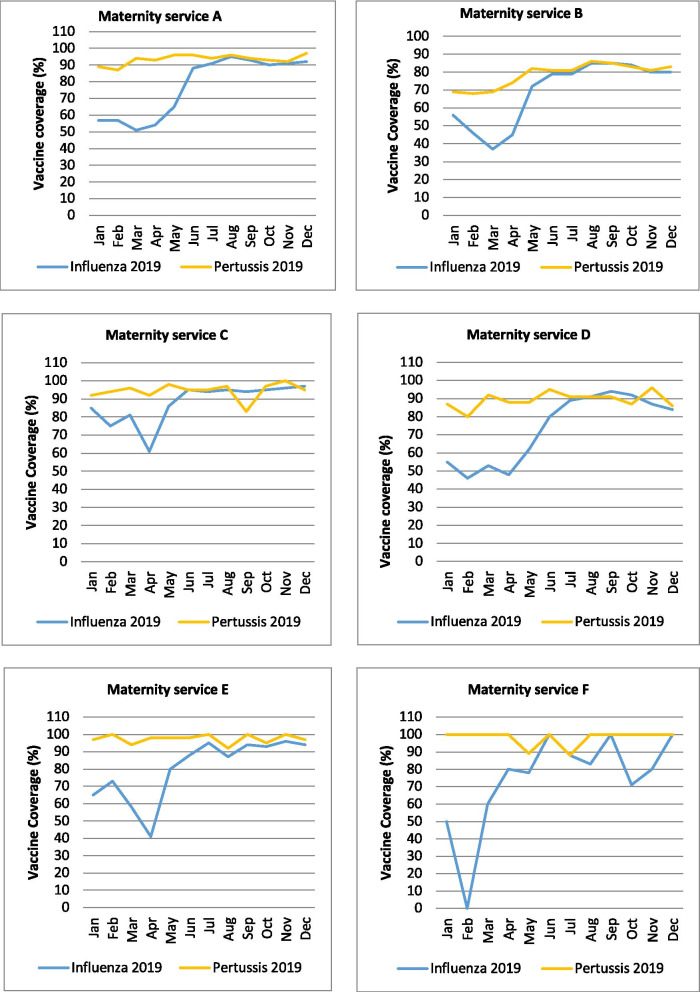
Influenza and pertussis coverage 2019 per maternity service

The monthly influenza vaccine coverage (January 2019 to December 2019) in each maternity service compared to their monthly pertussis vaccine coverage is summarised in Fig. 3.

### Economic evaluation

The annual aggregate cost of the new maternal immunisation platforms was AU$109,160, inclusive of $8822 (8%) capital costs; $2672 (2%) non-capital costs; $4528 (4%) supply costs; and $93,138 (85%) workforce costs. This represents an annual incremental cost increase of $58,628 when compared to the cost of the existing platforms (a 116% increase).

Across the evaluation period, the participating health services provided a total of 4486 maternal influenza and pertussis immunisations. Of this total, there were 1882 (42%) influenza and 2604 (58%) pertussis immunisations. This represents an increase of 3685 immunisations compared to the baseline period.

All services reduced their average cost per immunisation under the new platforms due to efficiencies achieved in the delivery of maternal immunisations. This cost saving ranged from $9 to $71.

Hospital F recorded the greatest reduction in the average cost per immunisation (−$71, from $146 to $75 per immunisation) followed by Hospital C (−$23, from $67 to $43 per immunisation), Hospital E (−$10, from $54 to $44 per immunisation) and Hospital D (−$9, from $56 to $46 per immunisation). The pharmacy led model, recorded the lowest average cost per immunisation of $10. Some of the new models demonstrated greater cost-effectiveness compared to others. That is, some models had a lower additional cost per additional immunisation, presented as an ICER. When comparing the ICER’s of the new models, the cost per additional immunisation ranged from $24 to $45: Hospital C recorded the lowest cost per additional immunisation (ICER = $24) followed by Hospital E (ICER = $35), Hospital D (ICER = $37) and Hospital F (ICER = $45).

## Discussion

This project partnered with six maternity services, and was able to demonstrate increased vaccine coverage across all services, particularly with respect to influenza vaccine. This occurred irrespective of the size of the service, the capability level or whether regional or metropolitan and irrespective of the innovation chosen by each health service. This suggests that it was not the actual innovation itself but other factors that contributed to this. The key factors contributing to success included: 1. undertaking a thorough analysis of the existing systems and pathways at baseline upon which to build any future intervention 2. working closely with key local stakeholders to co-design the innovation which the maternity service had local ownership over 3. training “vaccine champions” to drive the project at a grass roots level and 4. ensure accurate, timely, mandatory data capture to monitor progress and identify gaps. Increased coverage occurred no matter the craft group of the immuniser (GP, midwife or pharmacist). Our project has demonstrated that with a service-by-service approach, ensuring equitable access to influenza vaccine and education of staff, it is possible to close the gap between pertussis (which often has higher reported uptake) and influenza vaccine coverage (which often has a lower reported uptake). Furthermore, our study has demonstrated that although the annual incremental cost increased, when the number of vaccines given was considered, all services reduced their average cost per immunisation under the new platforms due to efficiencies. In addition to this, the increased vaccine coverage overall may further decrease costs to the healthcare system by reducing burden of disease.

Important lessons learnt with this project include the following;

***“There is no ‘one size fits all’ model to improve coverage”.***

Improvements were seen in all six maternity services partnering on the project despite very different platforms for delivery of vaccine (GP, midwife, pharmacist). The most important factor to ensure engagement from local stakeholders was to involve them in the process, thoroughly evaluate what infrastructure and systems were pre-existing and then co-design the intervention with the service and the stakeholders who were then charged with implementation of the intervention. Solutions were often there. The challenge was getting people who are really busy and from different sections of the health service together to make it happen. Listening to these locally derived solutions rather than imposing outsider views was key.

***“Project ownership and vaccine champions”.***

Having a local vaccine champion was powerful. This did not have to be someone trained as a nurse immuniser although this was reported as extremely beneficial by sites that incorporated this into their new delivery platform. The nurse immuniser naturally became the vaccine champion and was able to provide encouragement, support and answer any questions from colleagues. Importantly, they also were often involved in the role of monitoring the implementation of strategies to improve vaccine coverage, and led the initiative to rectify any barriers that need to be addressed in implementation. Because they worked in the antenatal clinic they were able to understand any challenges to implementation that arose and come up with practical solutions to address these challenges.

***“Interventions are only as good as the quality of data”.***

In this project, data was essential to a). identify the problem of low coverage b). to monitor the success of the implemented strategy and c). to provide feedback to the staff working to improve vaccine uptake. The previous initiative to make vaccination a mandatory field in Victorian Perinatal Data Collection assisted with this process. We also learnt from this project the importance of “real-time” or at least semi-contemporaneous entering of vaccination status compared with post partum. Maternity services were able to provide us with monthly updates of vaccination coverage during the course of the project. This was extremely useful to identify gaps early and to provide positive feedback.

***“Understanding the local landscape is essential and can’t be done without site visits”.***

The landscape analysis had two components. One was interviewing key stakeholders and the other was a “walk through” which included observation and confirmation of the information provided by stakeholders at a grass roots level. This was extremely valuable as often the information provided by stakeholders did not reflect what was actually happening on the ground. For example, at one hospital all the key stakeholders reported that no maternal vaccines were given on site. However, after the walk through it became evident that low risk women were being walked to delivery suite where pertussis vaccine was kept in the vaccine fridge and were having it administered there as part of their antenatal care. This information was vital to the subsequent development of the new model for influenza but would have remained unknown if the two complimentary components of the landscape analysis were not completed. In all settings, site visits were invaluable.

There are several strengths to our study. Our study conducted granular evaluation of barriers and facilitators through site visits and conversations with the stakeholders at each service. This enabled a modified model of vaccine delivery and strategies that were site-specific and locally acceptable. It has also helped the project team to better understand the local situation and improve our rapport with the stakeholders. Furthermore, having real-time vaccine coverage data helped inform the progress of our effort to improve vaccine coverage. There are also recognised limitations to this study. While some services had striking improvements, there were no control (no-intervention) services. However, the average influenza vaccine coverage reported across all maternity services for the time period 2019–2020 was 74.6% (well below the average of the six services participating in this project). Our study was not designed to compare one model of vaccine delivery against another and did not include private maternity services. Due to time and resource limitations, the assessment of barriers and facilitators were not conducted with pregnant women and the impact (both positive and negative) on pregnant women was not assessed. In addition, some services introduced other measures at their own discretion such as internal audits to monitor progress or a locally produced video promoting vaccination. It was not possible therefore to evaluate how much each individual intervention contributed to the increase in coverage. However, the overarching finding was that designing a locally relevant model that addresses local barriers and engaging local partners are more likely to work synergistically to achieve the desired outcome and successful implementation.

## Conclusion

Improving maternal vaccine coverage is an integral part of improving maternal and child health. Our study demonstrated that there is no ‘one size fits all’ model of vaccine delivery. Future successful strategies to improve maternal vaccine coverage at other maternity services should be site specific, multifaceted, targeted at the existing barriers to maternal vaccine uptake, and heavily involve local stakeholders in the design and implementation of these strategies. The cost-effectiveness analysis indicates that an increase in maternal influenza immunisation uptake can be achieved at a relatively modest cost through amendment of maternal immunisation platforms.

## Data Availability

Vaccine coverage data are reported to the Victorian Perinatal Data Collection and reported annually. The coverage data that support the findings of this study were provided by the participating maternity services in a de-identified form to the researchers to track vaccine coverage. All authors had full access to the coverage data provided by the health services. Complete datasets, which are potentially identifiable, including birth outcomes and vaccine coverage, are available from Consultative Council on Obstetric and Paediatric Mortality and Morbidity, Victorian Perinatal Data Collection but restrictions apply to the availability of these data because the release of potentially identifiable information to any persons not listed in s.41 of the Public Health and Wellbeing Act is only permitted for the purpose of research. This potentially identifiable data was not accessed for the purpose of this project.

## Abbreviations

WHO: World Health Organization
HCP: Healthcare provider
FAQ: Frequently asked questions
ICER: Incremental cost effectiveness ratio
GPs: General practitioners
